# The impact of biotropic weather on the incidence and severity of aneurysmal subarachnoid hemorrhage: a single-center observational explorative study

**DOI:** 10.1007/s00484-025-02890-y

**Published:** 2025-03-14

**Authors:** Carolin Albrecht, Kathrin Graw, Victoria Kehl, Isabel Hostettler, Bernhard Meyer, Andreas Matzarakis, Maria Wostrack

**Affiliations:** 1 Department of Neurosurgery, Klinikum rechts der Isar, School of Medicine and Health, Technical University of Munich, Munich, Germany; 2 Human Biometeorology Unit, Center for Medical Meteorological Research, German Meteorological Service, Freiburg, Germany; 3 Institute for AI and Informatics in Medicine, Klinikum rechts der Isar, School of Medicine and Health, Technical University of Munich, Munich, Germany; 4 Department of Neurosurgery, Cantonal Hospital St. Gallen, St. Gallen, Switzerland; 5 Chair of Environmental Meteorology, University of Freiburg, Freiburg, Germany; 6 Democritus University of Thrace, Komotini, 69100 Greece

**Keywords:** aSAH = aneurysmal subarachnoid hemorrhage, Subarachnoid hemorrhage, Weather conditions, Intracranial aneurysms, Bio-synop classes

## Abstract

Controversy surrounds seasonal variations in aneurysmal subarachnoid hemorrhage (aSAH) incidence and course. Investigating weather effects associated with weather fronts may provide more insights. Meteorological parameters are grouped into classes describing biotropic weather conditions influencing human health. Known as bio-synop classes, they impact human health by influencing biological processes. Our study explores the relationship between these classes and aSAH occurrence and severity using data from our neurovascular center and the German national weather registry. Data from 605 aSAH patients treated between 2006 and 2021 was analyzed. The Kruskal-Wallis Test was used to assess the incidence and severity of aSAH, classified by Hunt&Hess (HH) grades across bio-synop classes. Additionally, we explored seasonal clustering of aSAH events. While no significant difference in ASAH incidence across specific bio-synop classes was observed (*p* = 0.165), class 4 “Low-pressure with cold air advection” was significantly associated with a higher incidence of poorer HH grades (*p* = 0.022). Further, we observed a decrease in aSAH cases in June in month-to-month comparisons (May vs. June *p* < 0.001). Although bio-synop classes may not directly affect aSAH incidence, they appear linked to aSAH severity. The reduction in June aSAH cases suggests a potential seasonal influence, indicating a complex interplay of environmental factors warranting further investigation.

**Supplementary Information:**

The online version contains supplementary material available at 10.1007/s00484-025-02890-y.

## Introduction

Aneurysmal subarachnoid hemorrhage (aSAH) is a devastating condition associated with high mortality and morbidity rates and occurring at an incidence of 7.9 per 100.000 person-years(Helsper et al. 2021). Around 50% of aSAH patients are younger than 50 years old, and about 30% die in the early days to weeks following the hemorrhagic event (Feigin et al. 2003; O’Donnell et al. 2010). Moreover, the majority of survivors experience long-term cognitive impairment or permanent disability(Hackett and Anderson 2000). Despite extensive research, the factors that trigger aneurysm rupture remain incompletely understood. It is well known that weather conditions may exert a notable influence on human health across multiple dimensions. Extremes in temperature, whether high or low, can impose physiological strain on the human organism. Notably, the most significant impacts of weather on health in context of weather sensitivity are observed not during consistently abnormal conditions, but during substantial weather changes (Klaus Bucher 1993a). These effects are most pronounced at the frontal boundary of warm air advection and the typically unstable stratified cold air at the rear of the low-pressure system. The least negative impact on human health is found within the high-pressure center, provided there is no concurrent thermal or air quality stress. In addition, an individual’s response to weather is significantly influenced by personal and environmental factors, such as general health, sleep deprivation, stress, as well as landscape, climatic, seasonal, and diurnal factors (Klaus Bucher 1993b; Zacharias 2015). In order to assess the cumulative health-related effect of changes in meteorological parameters, the German Meteorological Service (*Deutscher Wetterdienst*, DWD) developed a specific classification of the so-called “bio-synop classes” (bio-synoptical classes, (Klaus Bucher [Bibr CR5][Bibr CR8]b). The current classification consists of five bio-synop classes: Class 1 denotes a stable high-pressure situation with mostly positive effects on health, Class 2 indicates warm air advection, Class 3 the center of a cyclone, occlusion- fronts als well as undulating fronts and Class 4 connotes cold air advection. Bio-synop classes 2,3 and 4 have the most negative effects on health. If none of the four classes can be assigned, it is defined as an indifferent weather situation (class 5, (Klaus Bucher 1993a).

Previous studies linked specific bio-synop classes respectively biotropic weather conditions to acute health disorders (Bobrovnitskii et al. 2014; Breuer et al. 1984; Klaus Bucher 1993a; Graw et al. 2022; Walach et al. 2002) including acute vascular conditions, such as aortic dissection (Kurz et al. 2024). Moreover, the incidence of asthma or chronic obstructive airway disease exacerbations and headaches is increased during bio-synop classes 2, 3, and 4 (Klaus Bucher 1993a). These three bio-synop classes reveal a negative association with numerous additional illnesses or symptoms, including exacerbations such as phantom pain, cramps, or colic. A recent study also found an increased risk of stroke-related hospitalization associated with sharp temperature changes and exposure to heat during the summer (Hao et al. 2024).

However, the potential impact of bio-synop classes on aSAH has not been previously explored. Our study seeks to investigate this matter using our large consecutive dataset.

## Methods

### Study design and patient cohort

**Fig. 1:**
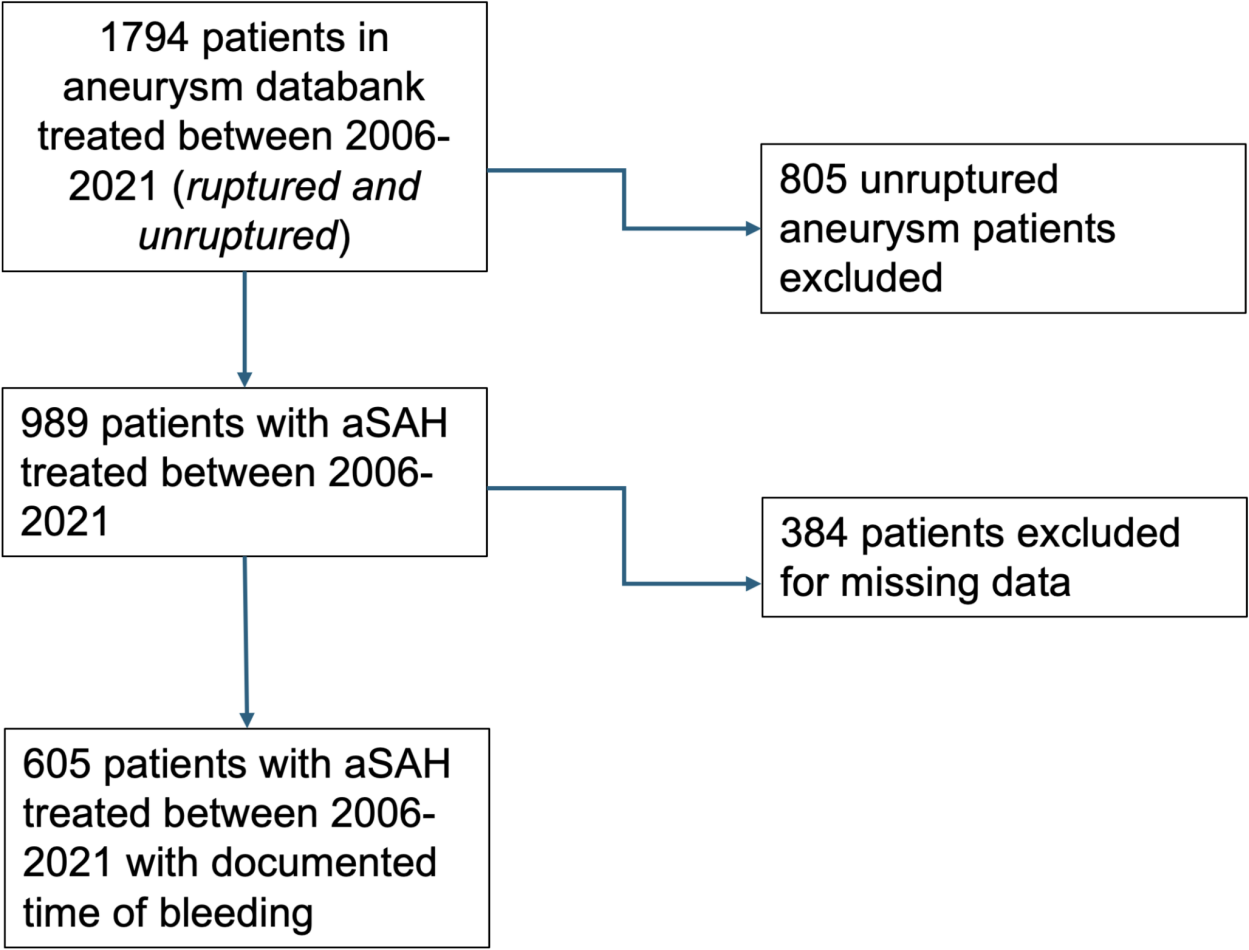
Flow chart for patient selection

The retrospective analysis involved prospectively collected data from our hospital-based registry of consecutive aSAH patients treated between January 2006 and November 2021 at our institution. Patients with unruptured intracranial aneurysms, perimesencephalic aSAH, or non-aneurysmal aSAH were excluded from the study, as were those with aSAH secondary to underlying pathologies such as mycotic, AVM-associated, dissection-related aneurysms, or pseudoaneurysms (*n* = 805). From the data of 989 consecutive patients with aneurysmal aSAH, only those with a known time of ictus were included (*n* = 605). aSAH onset time was determined either from the documented emergency call or, if not recorded, from the initial computed tomography (CT) scan, as described previously (Frontera et al. 2006). Patients for whom the initial timepoint of aneurysm rupture could not be determined with certainty within a 12-hour window were excluded from the study. The severity of the initial bleeding incident was categorized using the Hunt & Hess (HH) grading system (Hunt and Hess 1968). Patients with unknown clinical data on the initial HH grade, such as those who were treated in external hospital and referred to our center during the later clinical course, were also excluded from the analysis (*n* = 384, Fig. 1). The Fisher CT score was used to stratify the amount of SAH on the initial CT scan and thus served as an additional marker for the severity of the initial bleeding incident (Fisher et al. 1980). The study was designed in accordance with the STROBE guidelines (von Elm et al. 2007) and conducted in accordance with the principles of the Declaration of Helsinki. Informed consent was not required due to the retrospective nature of the analysis. The study was approved by the Research Ethics Committee of the Technical University of Munich (reference number 186/20S).

### Diagnostics and treatment policy

Our high-volume university vascular center, located in Munich, the capital city of Bavaria, serves the majority of Southern Bavaria within an approximate radius of 80 km. As neurovascular clinical care is a primary focus of our clinic, all aSAH patients referred to us by the regional dispatch center or other secondary care centers in the catchment area, are admitted and treated, regardless of the open capacity of intensive care and general ward beds at the time of admission.

Patients undergo digital subtraction angiography (DSA) and subsequent aneurysm occlusion within 24 h of admission, following our standard internal protocol. Decision on the treatment modality (endovascular vs. surgical) is made through interdisciplinary consensus between the senior representatives of the vascular divisions of the Neuroradiological and Neurosurgical Departments. In cases where patients exhibit clinical and/or imaging signs of markedly elevated intracranial pressure (ICP), such as large intracerebral hematoma or non-reactive pupils with elevated ICP measurements, ultra-emergent surgical clipping is performed, along with decompression procedures (hemicraniectomy, hematoma evacuation), without preceeding DSA.

### Bio-synop classes

**Fig. 2:**
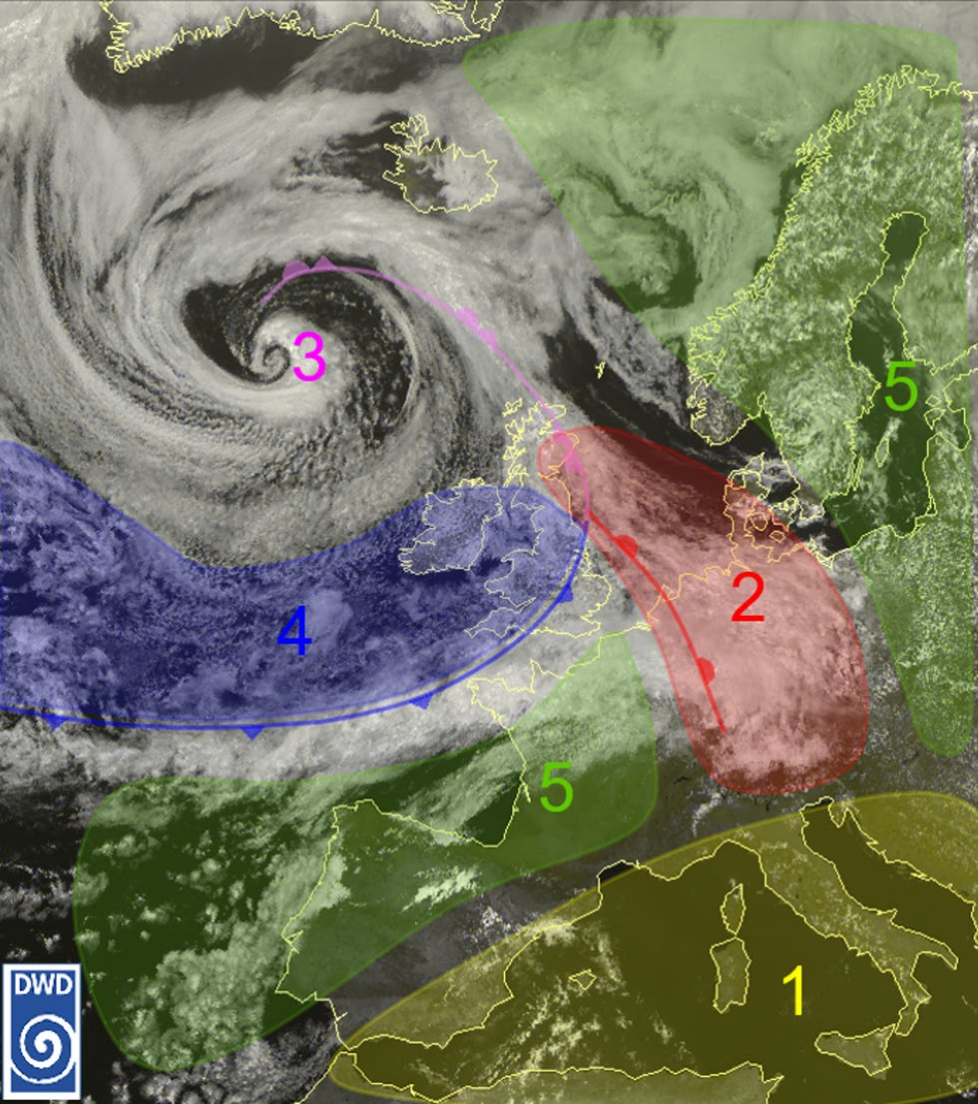
The five bio-synop classes

We used the established classification system of DWD (Fig. 2) to categorize weather conditions from a bio-synoptical perspective (Klaus Bucher [Bibr CR5][Bibr CR8]b).

The bio-synoptical classification is based on statistical correlations extracted from epidemiological investigations, enabling the precise characterization of the impact of meteorological factors on human health (Klaus Bucher 1993a). The five bio-synop classes are routinely used to predict potential health effects in weather-sensitive individuals (Graw et al. 2022).

These bio-synop classes encompass three parameters: the first describes the general weather situation with regions naturally occurring at a cyclone, a high pressure system or inbetween, the second reflects the magnitude and fluctuation of weather conditions, and the third quantifies thermal stress. In this study we focussed on the first parameter of the bio-synop classes.

The forecasts of bio-synop classes by the DWD are available for the current day and the following two days for 11 regions in Germany, segmented into two parts of each day. For this investigation, the daily occurrences of bio-synoptic classes within the hospital service catchment area (southern Bavarian region) from March 2006 to November 2021 were analyzed, divided into two time periods: 0–12 a.m. and 12–24 p.m. The meteorological data (bio-synop classes) was provided by the DWD.

### Statistical analysis

The study aimed to investigate two primary hypotheses: first, whether the occurrence of aSAH and second, its severity, as described by HH grades, are influenced by specific bio-synop classes. Additionally, a secondary analysis was conducted to explore potential seasonal clustering of aSAH occurrences. Furthermore, the association between aneurysm rupture and foehn wind, a distinct dry, warm, downslope wind commonly observed in southern Bavaria, was examined, given its presumed impact on health (Ficker and Rudder 1948; Rohden 1933).

Descriptive statistics included absolute and relative frequencies for categorical variables and median with range for continuous variables. The latter was preferred due to the skewed distributions. Differences were assessed using the Kruskal-Wallis test in case of ordinal data and Chi-square test in case of nominal data. All tests were performed two-sided at the 5% significance level. Due to the explorative nature of the study, no adjustment for multiple testind was done. P-values which would have been significant with more conservative significance levels of 1% or 0.1% are marked with [*] and [**]. Data analysis was conducted with IBM SPSS Statistics (version 28.0, Armonk, NY).

## Results

**Fig. 3:**
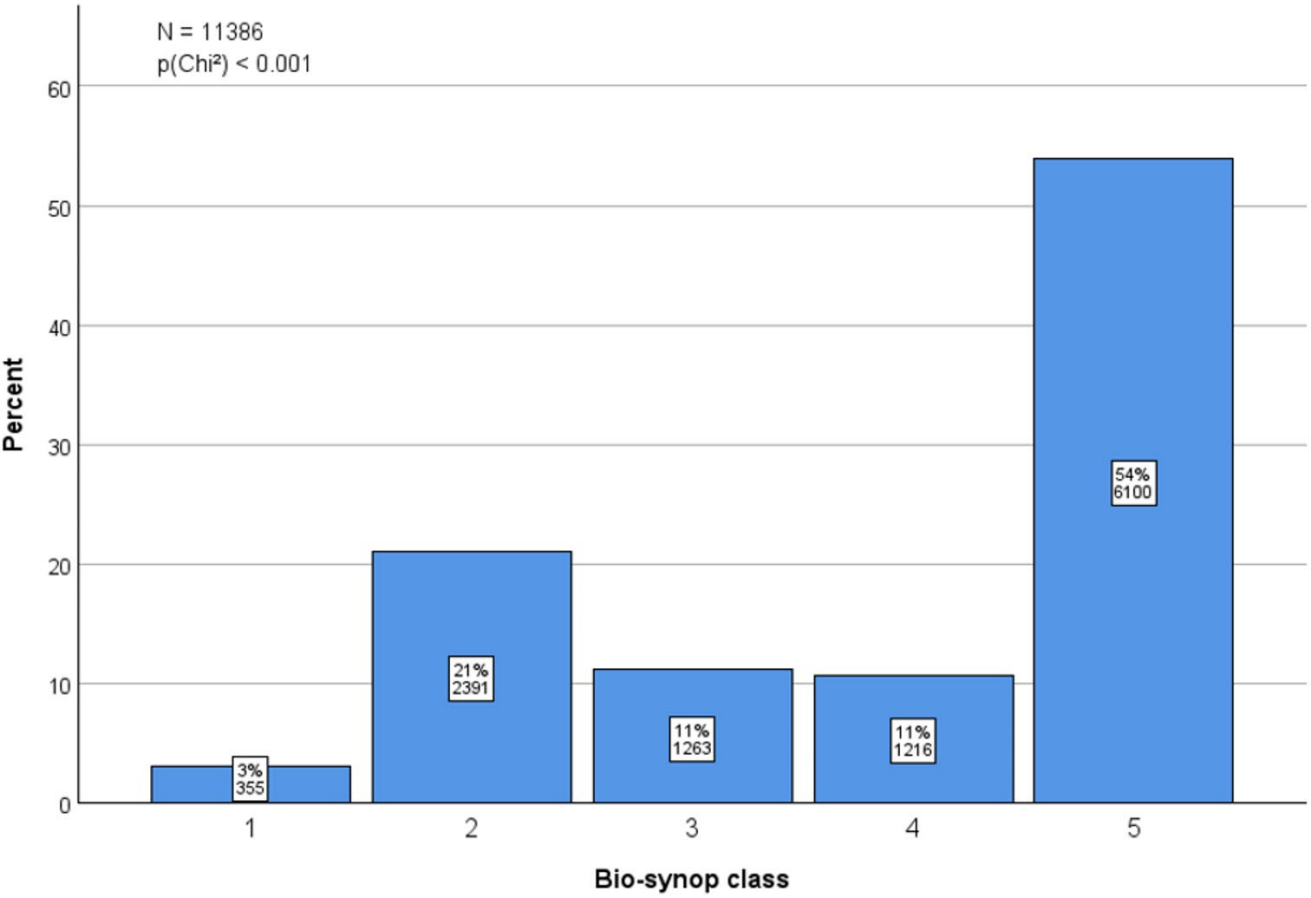
Distribution of five bio-synop classes within the service area of the study center during the investigated time frame (2006–2021)

In total, 605 patients were included to the analysis, comprising of 197 (32.6%) males and 408 (67.4%) females, with the median age of 55 years (range 21–93 years, Table 1). During the investigated period, the distribution of different bio-synop classes within the hospital service region consists of the most frequently observed class 5 with about 50% and the classes 1 to 4 all together with about 50%, too (*p* < 0.001) (Fig. 3).

**Table 1:** Patient demographics

	Patients with aSAH March 2006– November 2021 (*n* = 605)
Age (years), *median (range)*	55 (21–93)
Gender, *n (%)*	
Female	408 (67.4)
Male	197 (32.6)
H&H score, *median (range)*	3 (1–5)
H&H score, *n (%)*	
1	79 (13.1)
2	200 (33.1)
3	133 (22)
4	99 (16.4)
5	92 (15.2)
Fisher score, *median (range)*	3 (1–4)
Fisher score, *n (%)*	
1	20 (3.3)
2	48 (7.9)
3	459 (75.9)
4	38 (6.3)
Treatment, *n (%)*	
Clipping	228 (37.7)
Endovascular	354 (58.5)
Both	5 (1)
No treatment	15 (2.6)
Aneurysm location, *n (%)*	
ACA, AcomA	227 (37.5)
Posterior circulation	152 (25.1)
MCA	153 (25.3)
ICA	66 (10.9)

### Bio-synop classes and occurrence of aSAH

**Fig. 4:**
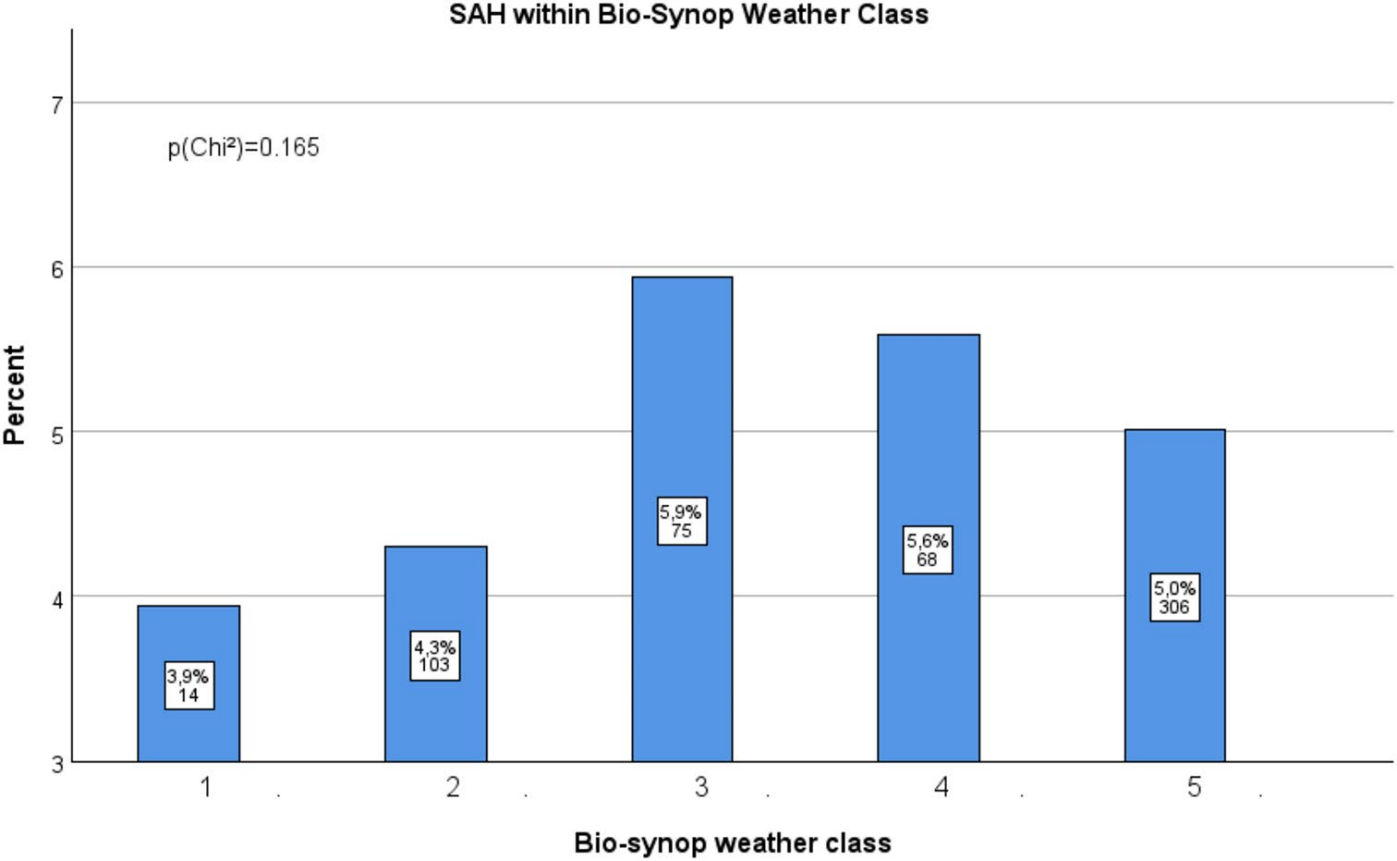
Association between the bio-synop classes and the occurrence of aSAH events. The graph shows percentage of the 12-hour periods during which patients with aSAH were admitted to the hospital. The total number of 12-hour periods was 11,386

The occurrence of aSAH was distributed almost equally across the different bio-synop classes. The Chi-square test was not significant at the 5% level (*p* = 0.165, Fig. 4). Although statistical significance was not attained, we have observed a trend toward aSAH events occurring more frequently, particularly at percentages of 5.9% and 5.6% during biosynop classes 3 and 4, respectively, known for their negative health effects; conversely, aSAH events appear less frequently at 3.9% during biosynop class 1, associated with positive health outcomes.

### Bio-synop classes and severity of aSAH

**Fig. 5:**
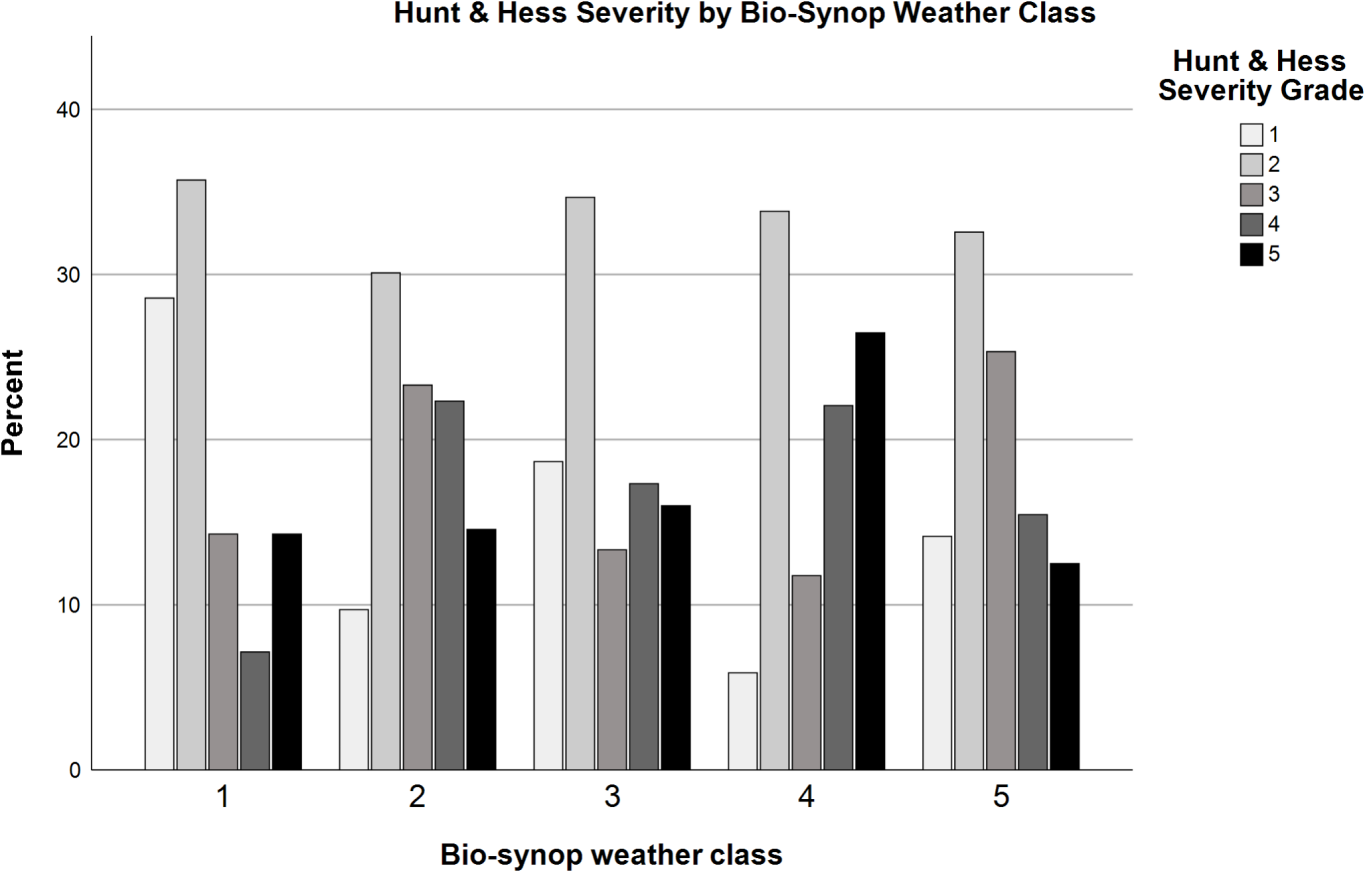
HH grade distribution across the different bio-synop classes

**Fig. 6:**
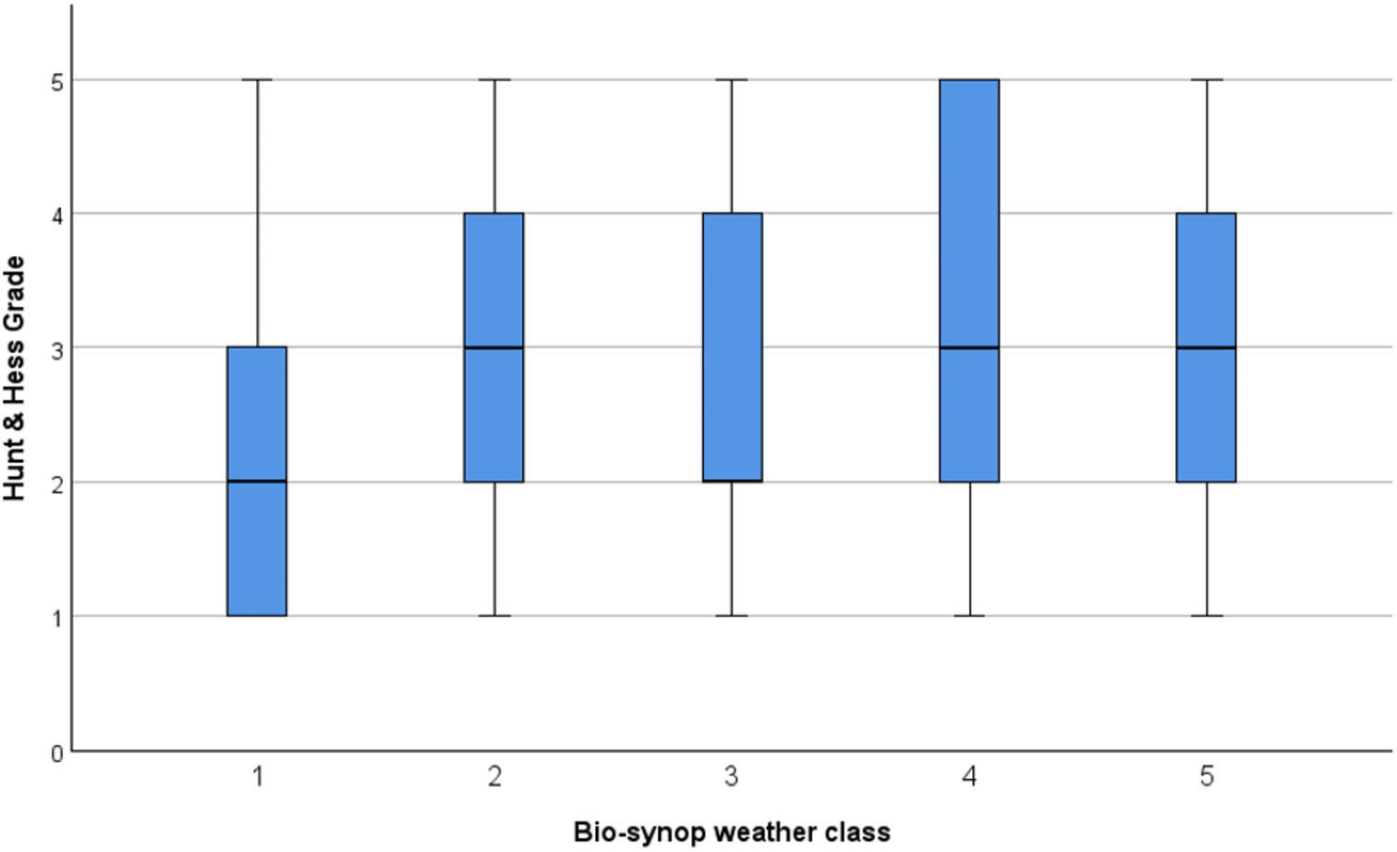
Distribution of HH within bio-synop (weather) classes

Within the examined cohort, we observed a clustering of milder versus more severe aSAH events during specific bio-synop classes. In particular, during bio-synop class 4 (“rear side of low-pressure system with cold air advection”), severe aSAH (HH grades 4 and 5) were significantly more frequent (*p* = 0.022). The individual absolute and relative frequencies are displayed in Figs. 5 and 6. Additionally, although not statistically significant, Fig. 5 suggests that the least severe HH grades tend to occur more frequently during bio-synop class 1, which has been previously described as having positive effects on health (K. Bucher and Haase 1993).

### Seasonal clustering

**Fig. 7:**
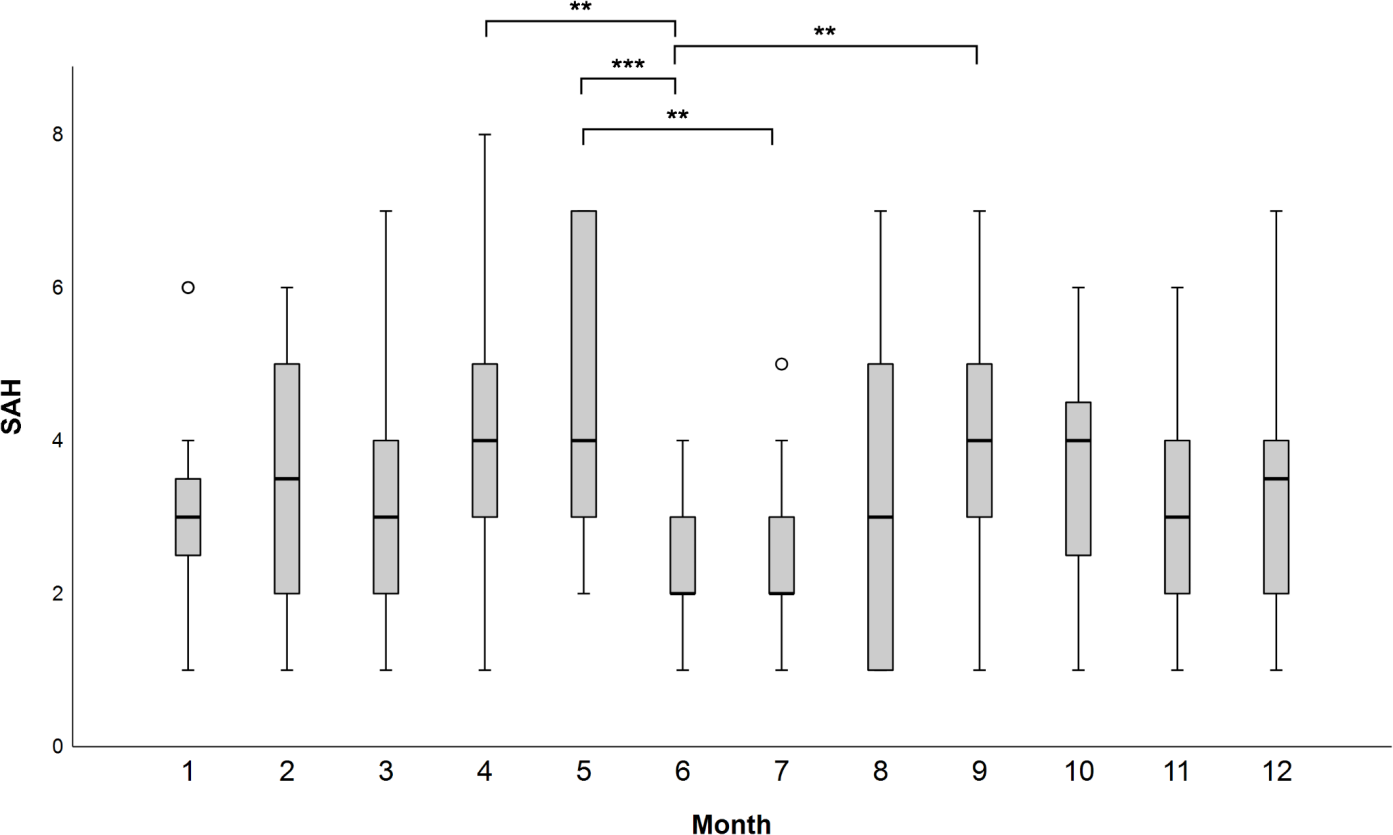
Seasonal clustering of aSAH events: Monthly variation in the number of aSAH events. Selected pairwise explorative tests: ** significant at the 1% level (*p* < 0.01); *** significant at the 0.1% level (*p* < 0.001). No significant correlation between the occurrence of foehn wind and aSAH events were observed (*p* = 0.205)

Regarding overall seasonal clustering of aSAH events, we observed a consistent decrease in aSAH cases in summer, and specifically in June, with significance achieved in subtests for specific months (*p* < 0.01), although the overall Kruskal-Wallis test was only nearly significant (*p* = 0.050; Fig. 7).

**Fig. 8:**
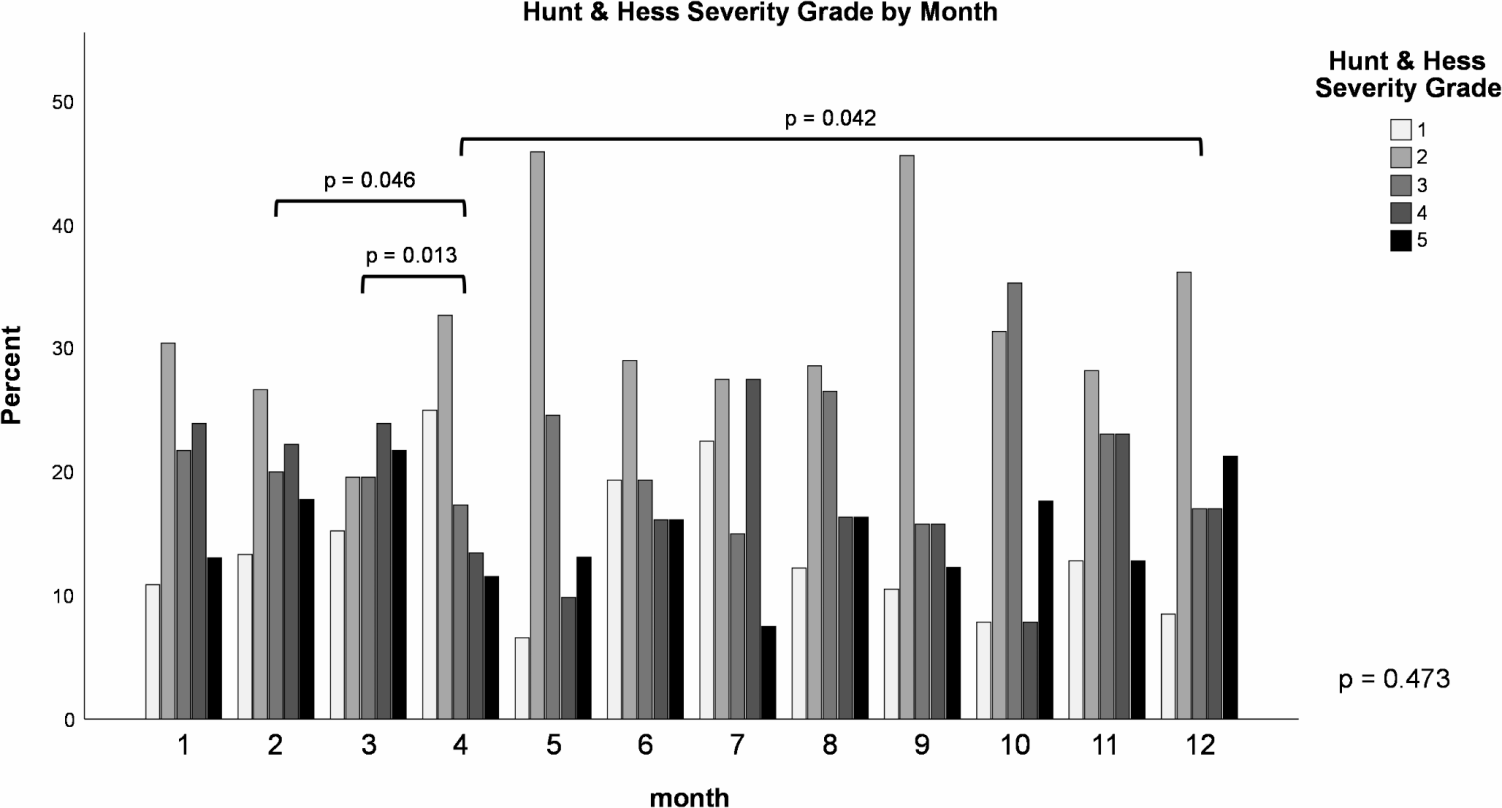
aSAH severity as measured by HH grades by calendar month. Kruskal-Wallist test for overall difference including selected explorative pairwise comparisons

Regarding aSAH severity, the overall comparison did not reach statistical significance. However, some individual month-to-month comparisons showed significant results, although these findings are considered exploratory (Fig. 8).

## Discussion

Numerous previous studies have explored weather-related factors associated with aneurysm rupture. However, most authors have primarily focused on overall seasonal variability in aSAH occurrence or isolated parameters such as temperature variations, atmospheric pressure, or humidity (Backes et al. 2016; Fukuda et al. 2019; Gill et al. 2013; Muroi et al. 2004). Controversial results have been reported, with some studies identifying low ambient temperature as the most powerful influence on aSAH (Backes et al. 2016; Kellogg et al. 2017; Yao et al. 2020), while others completely rejected this finding and proposed alternative influencing factors such as increased atmospheric pressure (Kockler et al. 2021). Recently, a German study employed Deep Learning models to analyze nearly all available meteorological parameters, including wind speed, rain- and snowfall, sunshine hours, cloud coverage, temperature, and atmospheric pressure; the results ruled out all of the previously assumed correlations between the aSAH and specific weather factors dismissing them as completely irrelevant for this disease(Helsper et al. 2021).

To our knowledge, our study is the first to report on the significance of bio-synop classes in aSAH. The key advantage of utilizing bio-tropic weather classes lies in their ability to capture a broader range of meteorological factors, derived from extensive previous research on their relevance for human health, in a more integrated manner, providing a more comprehensive understanding of their impact.

We observed a clear correlation between the HH grades and bio-synop classes: Class 4, characterized by the back of cold fronts with decreasing temperature and simultaneously increasing air pressure, was associated with a significantly increased severity of aSAH events. The reason for this association is not fully clear. Several explanations can be proposed. Class 4 is known to be a hypertension-favoring weather condition (Modesti et al. 2006). A study of Kurz et al. reporting on the relevance of acute aortic dissections has also highlighted the determining link to bio-synop class 4, similarly to our findings, particularly due to its association with hypertension-related changes (Kurz et al. 2024). Cold exposure is known to trigger sympathetic activation, elevate systemic vascular resistance, thereby increasing central aortic blood pressure (Vaiciulis et al. 2023). Increased blood pressure following the aneurysm rupture may potentially increase the likelihood of a more pronounced bleeding preceding the subsequent aneurysm wall repair. Individuals with higher blood pressure levels during the onset of aneurysm rupture may therefore experience more severe clinical symptoms (Rosengart et al. 2007). Moreover, cold weather fronts have been associated with pro-thrombotic features, suggesting a potential contribution to impaired perfusion and acute (micro)vascular damage, as proposed in analogous biometeorological research on ischemic stroke (Lavados et al. 2018). Additionally, potential activation of proinflammatory pathways has been proposed (Vaiciulis et al. 2023).

Contrary to our initial hypothesis, we found no statistically significant correlation between aSAH occurrence and bio-synop tropic classes, indicating that the risk of rupture is likely dependent on factors other than those encompassed in meteorological conditions encapsulated in bio-synop classes. Additionally, the occurrence of foehn winds, often believed to play a significant role in various acute illnesses, particularly according to general practitioners and popular belief (Ficker and Rudder 1948; Rohden 1933), showed no relevance for aSAH.

Furthermore, we observed a clear trend towards a lower number of aSAH events in summer, particularly in June, although significance was reached only in subgroup analyses. These findings align with previous research, as the majority of studies have reported lower aSAH rates in summer compared to other seasons (Backes et al. 2016; de Steenhuijsen Piters et al. 2013; Han et al. 2017; Kellogg et al. 2017; Nyquist et al. 2001). The prevailing explanation for this phenomenon has been the warmer air temperatures in summer and vice versa, with cold winter weather accelerating the risk of aneurysm rupture (Backes et al. 2016; Han et al. 2017; Kellogg et al. 2017). More recent research, however, states that significant changes in host’s humoral and cellular immune functions occur in seasonal patterns, and this happens independently of temperature fluctuations (Bratescu and Teodorescu 1981; Fares 2013). This may also explain, why specific bio-synop classes, which include air temperature as one of the determining parameters, did not significantly impact aSAH risk in our cohort. Instead, the seasonality of aneurysm ruptures appears to be more likely attributed to a complex interplay between the immune activation of individual organisms and various environmental factors, which extend beyond established meteorological parameters. While there are no direct preventive measures that can specifically prevent aneurysm rupture within each Bio-Synop class, the available data may increase sensitivity in the early detection of symptoms associated with aSAH, thereby facilitating earlier diagnosis and initiation of treatment. In addition, the present data will allow for the development of a risk prediction model that will promote increased awareness of the disease.

Future studies focusing on seasonal immunology in aSAH are warranted, as they could provide new insights into the underlying mechanisms and help to refine our understanding of the environmental effects on aneurysm rupture.

### Limitations

The retrospective design of the study inherently carries known disadvantages, including potential biases in data collection as well as potential incomplete or inaccurate patient information. The retrospective design was chosen due to practical constraints, as it allowed us to efficiently analyze a large dataset spanning over a significant timeframe, without the need for additional data collection, reducing the burden on resources and time. Even already within our own database, some entries had to be excluded due to incomplete recordings of the exact time of bleeding events, potentially introducing selection bias. A significant limitation is that a part of aSAH events occurring within the capture area were, were possibly treated in other hospitals or neighboring regions, leading to incomplete data and hindering reliable result interpretation. Additionally, a certain rate of aSAH events was not reported, particularly for severe aSAH cases, as some patients may not have reached the hospital and were thus not included in the registry, which may skew the true incidence of aSAH.

While we did not perform a detailed geospatial analysis to track potential shifts in the catchment area over time, our hospital’s status and role as a primary referral center remained unchanged throughout the study period. Future studies could incorporate geospatial mapping of patient origins to confirm the stability of the catchment area and assess potential variations over time.

Our data is subject to another selection bias, as the observed seasonal variation in the number of referrals at a single center in this study may simply reflect the seasonal activity patterns of different hospitals. To accurately investigate meteorological factors, the study should include all patients within the defined area.

Furthermore, the study lacked any information on potential confounding variables such as comorbidities, lifestyle factors, and individual exposure to specific bio-synop classes or other environmental factors, which could influence the observed connections to aSAH outcomes. While the large sample size and extended study period provide a comprehensive overview of aSAH occurrence in the region, caution is warranted when generalizing the results to the broader population.

## Conclusion

Our study examined the connection between bio-synop classes and the occurrence and severity of aneurysmal subarachnoid hemorrhage (aSAH). Analyzing data from 605 patients, we found no significant correlation between aSAH incidence and specific bio-synop classes. However, our results indicate a significant association between the bio-synop class 4 (rear side of low-pressure system with cold air advection), and increased severity of aSAH cases. Additionally, a notable seasonal trend was observed, with a decrease in aSAH incidents during June, suggesting a potential seasonal influence on aSAH occurrences. These findings highlight the complex interplay between environmental factors and aSAH, emphasizing the need for further research to understand these relationships fully. Understanding these dynamics is crucial for developing more effective predictive models and prevention strategies for aSAH.

## Electronic supplementary material

Below is the link to the electronic supplementary material.

Supplementary Material 1

## Data Availability

The source data are securely stored at the Department of Neurosurgery, Technical University of Munich, Germany. To access the raw data or to discuss potential collaboration, please contact the corresponding author directly.
