# Supplementary material for: The impact of biotropic weather on the incidence and severity of aneurysmal subarachnoid hemorrhage: a single-center observational explorative study
Source: Int J Biometeorol. 2025 Mar 14;69(6):1267–77. doi: 10.1007/s00484-025-02890-y (PMC12141114; doi:10.1007/s00484-025-02890-y)
Supplement: Supplementary file 1 — Supplementary Material 1 [file 484_2025_2890_MOESM1_ESM.docx]

**Supplementary Material:**

| Bio-synop class | Did SAH events occur? | | | | |
| --- | --- | --- | --- | --- | --- |
|  | No | | Yes | | Total |
|  | N | Row % | N | Row % | N |
| 1 | 341 | 96.1% | 14 | 3.9% | 355 |
| 2 | 2288 | 95.7% | 103 | 4.3% | 2391 |
| 3 | 1188 | 94.1% | 75 | 5.9% | 1263 |
| 4 | 1148 | 94.4% | 68 | 5.6% | 1216 |
| 5 | 5794 | 95.0% | 306 | 5.0% | 6100 |
| Missing | 61 |  | 0 |  | 61 |
| Total | 10820 | 95.0% | 566 | 5.0% | 11386 |

**Table 2.** Absolute and relative frequencies of 12-hour periods with and without SAH events.
